# Complementary Proteomics, Genomics approaches identifies potential pathogenicity/virulence factors in *Tilletia indica* induced under the influence of host factor

**DOI:** 10.1038/s41598-018-37810-1

**Published:** 2019-01-24

**Authors:** Vishakha Pandey, Atul Kumar Gupta, Manoj Singh, Dinesh Pandey, Anil Kumar

**Affiliations:** Department of Molecular biology and Genetic Engineering, G.B. Pant University of Agriculture and Technology, Pantnagar, Uttarakhand India

## Abstract

Karnal bunt disease of wheat is incited by quarantine fungal pathogen *T*. *indica*. Till date, there is little information on the pathogenic mechanisms involved in Karnal bunt. In order to understand the molecular mechanisms of disease pathogenesis, highly aggressive *T*. *indica* TiK isolate was cultured in the presence of host factor extracted from developing spikes of wheat variety WH-542. Modulation in protein profile of mycelial proteins and secretome from TiK cultured in the absence and presence of host factor was analyzed by 2-DE. Fifteen and twenty nine protein spots were up-regulated/differentially regulated in the proteome of mycelial and secreted proteins, respectively and identified using MALDI-TOF/TOF. Identified proteins are involved in suppression of host defense responses, lignin degradation of plant cell wall, penetration, adhesion of pathogen to host tissues, pathogen mediated reactive oxygen species generation, hydrolytic enzymes, detoxification of host generated reactive oxygen species. Further, integration of proteomic and genomic analysis has led to candidate pathogenicity/virulence factors identification. They were functionally annotated by sequence as well as structure based analysis. In this study, complementation of proteomics and genomics approaches resulted in novel pathogenicity/virulence factor(s) identification in *T*. *indica*.

## Introduction

Wheat (*Triticum aestivum*) is the most widely cultivated crop, covering 17% of the world’s acreage **(**https://www.idrc.ca/en/article/facts-figures-food-and-biodiversity**)**. Wheat is a rich source of carbohydrate, dietary fibers, minerals, proteins and vitamins and provides more calories to the world’s diet than other crops. Wheat productivity has not kept pace with the increased world population. By year 2050 the world’s population is estimated to reach 9.6 billion, it is expected that global wheat production will need to increase upto 60%, in order to meet the increasing demand **(**www.igc.int**)**. Being staple food for 40% of the world’s population, there is always a high demand for wheat which drives the growth in wheat cultivation.

Wheat production is severely affected by various fungal diseases such as rusts, molds, smuts, blights, bunts and mildews. Among them, Karnal bunt (KB) disease incited by hemibiotrophic smut fungus *Tilletia indica* partially damages and replaces the kernels with black powdery mass of fetid spores. Since only a portion of the kernel is damaged, the disease is often known as partial bunt. It was named after Karnal city, India from where it was first recorded in 1931. The disease has also been reported from several countries such as South Africa, Mexico, Nepal, Arizona, USA, Iran, Afganistan, Iraq, Brazil and Pakistan **(**https://www.cabi.org/isc/datasheet/36168**)**. KB not only reduces the crop yield but also the seed quality and germination potential. Wheat seeds with more than 1% infected seeds becomes inedible and wheat lots having more than 3% bunted grains should not be used for consumption by humans, This downgrades a good quality wheat seeds to animal feed, causing huge financial losses to the wheat producers. *T*. *indica* has been considered as quarantined pest in 70 countries and thus hindered the free movement of wheat consignments to countries that are currently free from KB pathogen^1^.

Plant pathogenic fungi utilizes various pathogenicity/virulence factors for infecting their hosts. They are usually classified as toxins effective against host defences, parts of signal transduction machinery, transporters for defending the pathogen against host defence system, penetration effectors and enzymes involved in degradation of host defenses^2^. The pathogenicity/virulence factors may be utilized as biomarkers for KB resistant wheat variety screening and development of *T*. *indica* diagnostics.

The floret infecting pathogen, *T*. *indica* can attack at distinct developmental stages of host, ranging from boot stage to partial spike emergence, boot stage to full spike emergence, boot stage to anthesis or at anthesis^3–5^. However, wheat exhibit different degree of susceptibility to infection at different developmental stages, indicating a direct relationship between the developmental stages of wheat and *T*. *indica* infection. In our laboratory, different extracts such as aqueous, salt, acetone and methanol, prepared from different parts of wheat (i.e. stem, leaf, S-1, S-2 and S-3 stages of inflorescence) were added to the growth media and morphogenetic development of KB pathogen was studied in terms of fungal biomass and radial growth. It was observed that mycelia growth was induced when fungus was cultured in the media containing acetone extract prepared from the S-2 stage of inflorescence^6^. The comparative study on the susceptibility of wheat to infection by *T*. *indica*, demonstrated that spikes at boot emergence stage (S-2) were more susceptible than other stages^7^. The effect of host factor on the expression of MAP kinase signaling machinery components, namely TiPmk1, TiFus3 and TiKpp2 (homologs of *U*. *maydis* Pmk1, Fus3 and Kpp2, respectively) was studied in our laboratory. The host factor induced the expression of Mitogen activated protein (MAP) kinase genes, indicating its role in fungal pathogenesis^8^. It also alters the genetic constitution of monosporidial lines due to change in protein expression^9^. To date, there is no report on the effect of host extract on the proteome of *T*. *indica*. The present study was conducted with the objective to mimick the *in vivo* host-pathogen interaction and analyze the modulation in protein profile of mycelia proteins and secretome from *T*. *indica* highly aggressive TiK isolate cultured in the presence of host factor using mass spectrometry based proteomics approach. Complementation of the results obtained by proteomic studies with *T*. *indica* genome information generated by the hybrid approach^10^ has led to putative pathogenicity/virulence factors identification in *T*. *indica*. They were further functionally annotated by sequence and structure based analysis.

## Materials and Methods

### *T*. *indica* isolates, plant material and culture conditions

Highly aggressive, Karnal isolate TiK was selected after evaluation of aggressiveness of ten isolates on ten *Triticum aestivum* differentials^11^. Isolate was cultured from a teliospore on potato dextrose agar (PDA) following the method previously described^12^ with alternating dark and light conditions for 15–20 days at 20 ± 2 °C in biological oxygen demand (BOD) incubator. Seeds of KB susceptible wheat variety WH-542 were planted at G.B. Pant University of Agriculture and Technology, Pantnagar for host factor extraction.

### Preparation of host factor/extract

Acetone extract was prepared from the spike of wheat variety WH-542 in S2 stage (boot emergence)^6^. Wheat spikes (50 g) were ground in pestle mortar using liquid nitrogen to a fine powder and subsequently suspended in chilled acetone (10 ml/g spikes) for 5 hours. It was filtered through muslin cloth and stored at 4 °C. Just before use, acetone was evaporated by blowing hot air at room temperature. About 1/10^th^ of the volume of sterilized ultrapure water was added to the obtained dried material. Prior to addition to culture media, it was filtered through 0.22 µ filter syringe filter.

### Extraction of mycelial and secreted proteins from TiK isolate cultured in the presence of host factor

TiK isolate grown in potato dextrose broth (PDB) in absence and presence of host factor was harvested through gravitational filtration using folded muslin cloth at 14^th^, 21^st^, 30^th^ and 40^th^ days after inoculation^11^. Growth curve was plotted using lyophilized biomass obtained at distinct time intervals. Culture supernatant was kept at −20 °C until utilized for secretory protein isolation.

Protein extract from TiK isolate cultured in presence and absence of host factor was obtained from lyophilized mycelia (0.5 g) following Vishakha *et al*.^11^ with slight modification. Sample was grinded and centrifuged (10,000xg, 20 min, 4 °C). Supernatant was collected and sonicated for 2 min (20 sec pulse on, 20 sec pulse off). Supernatant containing extracted mycelial proteins were precipitated by adding triple the volume of ice cold 10% TCA in acetone and incubated at −20 °C overnight. This is followed by centrifugation at 10,000 rpm at 4 °C for 15 min and washing the pellet using ice cold acetone. Final pellet was collected by centrifugation (10,000 × g, 10 min, 4 °C), air-dried and dissolved in solubilization buffer^11^.

Secretory protein was isolated following Vishakha *et al*.^13^ with slight modification. Culture filtrate was centrifuged (10,000 × g, 4 °C, 15 min,) to discard residual mycelia and polysaccharides. Supernatant was sonicated for 2 min (20 sec pulse on, 20 sec pulse off) for isolation of secreted proteins from TiK isolate cultured in the presence of host extract. This step is excluded for secreted protein isolation grown in the absence of host factor. Supernatant was transferred to fresh tube and thrice the volume of ice cold 10% TCA in acetone was added and incubated overnight at −20 °C. Secreted proteins were recovered following Vishakha *et al*.^13^.

Extracted mycelial and secreted proteins were quantified through Bradford method^14^.

### Two – dimensional electrophoresis, Gel analysis

2–DE was performed as previously described^13^. Concisely, 125 μg of protein was solubilized in IEF rehydration buffer. Passive rehydration of 7 cm IPG (immobilized pH gel) strip pH 3–10 (BioRad Laboratories, India) was carried out at room temperature for 16 h. Isoelectric focusing (IEF) was performed according to Fragner *et al*.^15^. After IEF, the IPG strips were reduced with equilibriation buffer I (6 M urea, 0.375 M Tris (pH 8.8), 130 mM DDT, 10% SDS and 20% glycerol) for 10 min. This is followed by alkylation with equilibriation buffer II (6 M urea, 0.375 M Tris (pH 8.8), 130 mM Iodoacetamide, 10% SDS and 20% glycerol) for 10 min. SDS-PAGE was conducted with 12% acrylamide gels using bromophenol blue (Merck Millipore, US) at 100 V. Gels were stained by Coomassie G-250 for 4 h and destained using destaining solution (10% methanol (v/v) and 7% glacial acetic acid (v/v)). Gel image was acquired in triplicates for each sample through alphaimager gel documentation and analyzed by IMP7 software. Spots that were differentially regulated and upregulated consistently in triplicate gels, were further identified using tandem mass spectrometry.

### MALDI-TOF/TOF analysis and database search

Spots were excised from the gels and destained in 50% methanol and 50 mM ammonium bicarbonate for 1 h at 40 °C. After complete dehydration, gel pieces were rehydrated in a solution containing 10% acetonitrile, 40 mM ammonium bicarbonate and 5 ng/µl of trypsin on ice bath for 30 min. This is followed by digestion for 16 h at 37 °C and extraction by 5% trifluoroacetic acid (TFA) in 50% acetonitrile solution. The peptides were further suspended in 1:1 in matrix solution (5 mg/ml of α-cyano-4 hydroxycinnamic acid, 50% acetonitrile and 0.1% trifluoroacetic acid), spotted onto MALDI target plates and MALDI-TOF/TOF was carried out. MS spectra obtained with 1600 laser shots per spectrum and tandem mass spectra was obtained with 2500 laser shots per fragmentation spectrum. Spectral analysis and peak list files were generated using Flex analysis software 3.0. Tandem mass spectra were searched against NCBIprot database version 20170707, 126069994 sequences and 46272342986 residues using MASCOT software **(**http://www.matrixscience.com**)**. Search parameters were as follows: enzyme, trypsin; taxonomy, fungi; no restriction of protein molecular weight; one missed trypsin cleavage; fixed modifications of cysteine (carbamidomethylation); variable modifications of methionine (oxidation). The mass tolerance for the peptides was 2 Da. When the score exceeded the homology threshold value calculated by MASCOT (p < 0.05), then the peptides were considered to be identified. The identified proteins were functionally annotated through Uniprot database **(**www.uniprot.org/**)** and categorized into different groups using Clusters of Orthologous Groups of proteins and Gene Ontology databases. TargetP^16^ was used to predict the subcellular localization of the identified proteins.

### Candidate pathogenicity/virulence factors identification from *T*. *indica* genome

Homologs of candidate pathogenicity/virulence factors were identified from the sequenced genome of TiK isolate^10^ by BLASTP. Identified pathogenicity/virulence factors were annotated using NCBI conserved domain database (CDD)^17^, InterProScan^18^, SMART^19^, ScanProsite^20^, CATH^21^ and, PANTHER^22^. 3-D protein models of identified pathogenicity/virulence factors were predicted by RaptorX software (http://raptorx.uchicago.edu) and validated using bioinformatics tools like RAMPAGE^23^, ProFunc^24^, DALI server^25^ and ProQ server^26^,

## Results

### Effect of host extracts on the growth kinetics of *T*. *indica* highly aggressive TiK isolate

**Figure 1:**
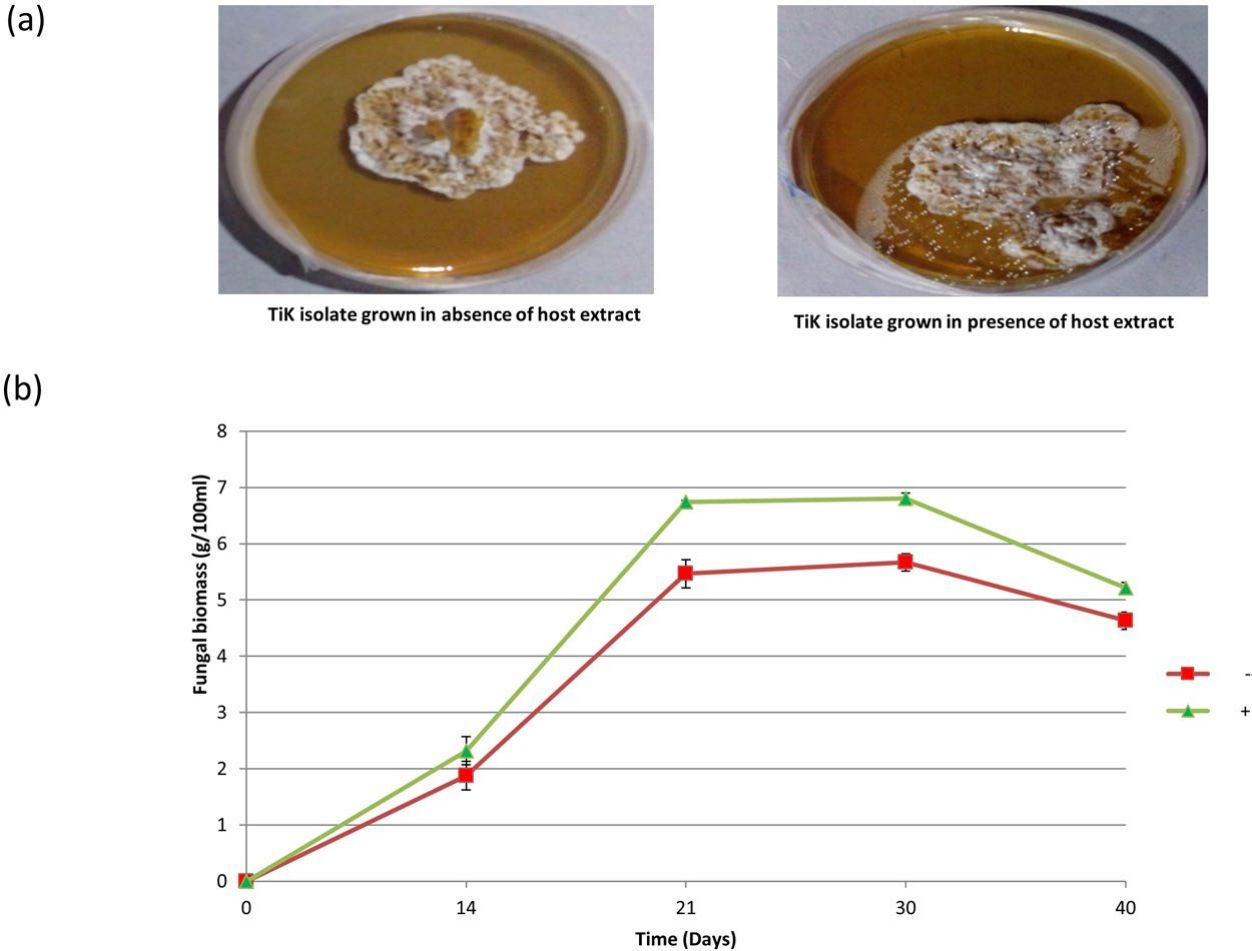
(**a**) Growth of *Tilletia indica* highly aggressive TiK isolate cultured in absence and presence of host extract (**b**) TiK isolate growth kinetics when grown in the absence (−) and presence (+) of host extract with respect to total biomass produced (g/100 ml of dry weight) at different time intervals.

The vegetative mycelium of *T*. *indica* exhibited exponential growth (logarithmic growth) upto 21 days, after which the growth rate declined (stationary phase) (Fig. 1b). At 14^th^ day (lag growth phase), the biomass was about 1.8 g/100 ml of fungal culture. It reached upto 5.4 g/100 ml at 21^st^ day (exponential phase) in the absence of host extract. However, the biomass was higher, approximately 2.3 g/100 ml of fungal culture at lag growth phase (14^th^ day) that increased upto 6.7 g/100 ml mass of mycelial mat at exponential growth phase (21^st^ day), in the presence of host extract (Fig. 1b). After 21^st^ day, a decrease in growth with respect to dry weight of fungal biomass was observed, both in the absence and presence of host extract. This is because of depletion in the nutrients available for growth. As shown in Fig. 1b, the growth rate of TiK isolate was much higher when grown in the presence of host extract.

### Comparative mycelial proteome analysis from TiK isolate grown in the presence and absence of host extract

**Figure 2:**
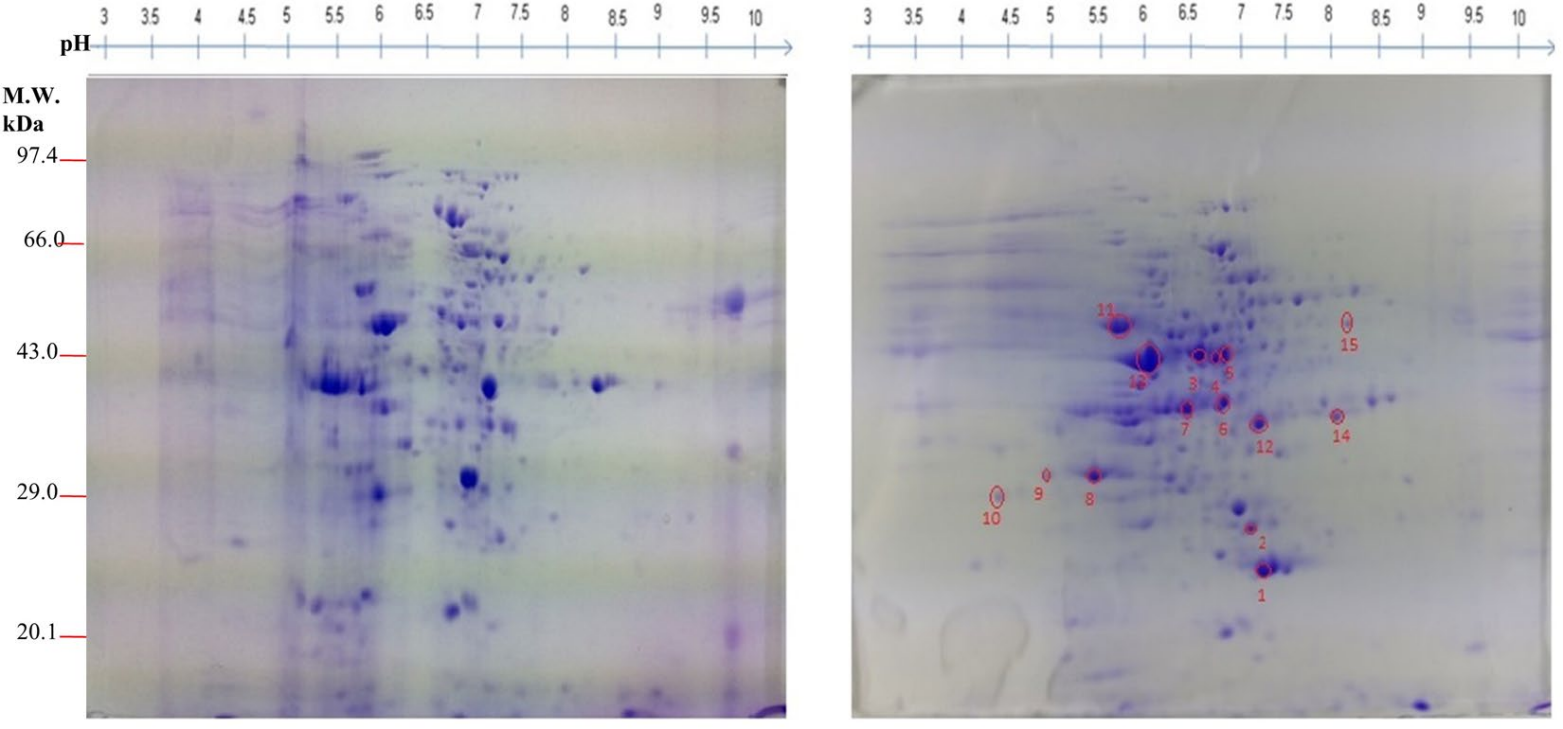
2-DE CBB stained gel of mycelial proteins from *T*. *indica* TiK isolate (**a**) cultured in the absence of host extract (**b**) cultured in the presence of host extract. Proteins were separated on 7 cm linear gradient IPG strips pH 3–10 and 12% polyacrylamide gel. Proteins spots exhibiting upregulation/differential regulation in TiK isolate (in the presence of host extract) are encircled red. Representative 2-DE gels are shown in Supplementary Figs S1 and S2.

Proteome map (pH 3–10) of the proteins extracted from TiK mycelia grown in the presence and absence of host extract was obtained (Fig. 2; Supplementary Figs S1 and S2**)**. The proteome profiles of the mycelial proteins extracted from TiK isolate cultured in the absence and presence of host extract were compared. Fifteen protein spots that showed 2.0 fold change in triplicate gels were considered as statistically significant (p < 0.05) and further identified by MALDI-TOF/TOF.

#### MALDI-TOF/TOF analysis and database search

Identfication of differentially regulated and upregulated proteins was carried out by MALDI-TOF/TOF. All the proteins identified have the well known functions. However, the proteins with spot number 2, 4, 10, 12, 13 and 15 were identified as hypothetical. The identified proteins have the theoretical molecular mass in the range from 16 to 394 kDa. The identified spots were encircled and numbered on the gels (Fig. 2). Table 1 shows the identity of putative proteins, matched peptides number and protein score.

**Table 1:** Identified up-regulated and differentially regulated proteins (pH 3–10) in highly aggressive *Tilletia indica* isolate TiK grown in the presence of host extract by MALDI-TOF/TOF.

Spot	Protein	Accession	Organism	Function	Exper. pI/Mr	Theo. pI/Mr	MASCOT Score	No. of Peptides
1	1,4-benzoquinone reductase	OAJ02041.1	*Tilletia caries*	Involved in lignin degradation.	7.25/22.35	6.70/22.00	141	4
2	Hypothetical protein	EYE93962.1	*Aspergillus ruber*	—	7.14/28.24	7.11/27.14	84	5
3	Salicylate hydroxylase	OJJ80335.1	*Aspergillus glaucus*	Degrade plant produced Salicylic acid and suppress plant defense response.	6.57/45.34	5.90/46.51	82	5
4	Hypothetical protein	EQK97475.1	*Ophiocordyceps sinensis*	—	6.75/45.33	6.60/45.32	82	10
5	S-adenosylmethionine synthetase	OAJ18635.1	*Tilletia walker*	Involved in development, stress response.	6.84/45.34	5.95/42.49	122	6
6	Isocitrate lyase	GAT26921.1	*Aspergillus luchuensis*	Involved in cuticle penetration and appressorium formation.	6.80/40.54	6.30/59.70	82	7
7	Isochorismatase hydrolase	KRH93850.1	*Verticillium dahlia*	Degrade Isochorismate, precursor of salicylic acid biosynthesis.	6.52/40.52	6.45/84.11	92	14
8	Isocitrate lyase	GAT26921.1	*Aspergillus luchuensis*	Involved in cuticle penetration and appressorium formation.	5.50/29.54	6.30/59.70	85	8
9	Isochorismatase hydrolase	KRH93850.1	*Verticillium dahlia*	Degrade Isochorismate, precursor of salicylic acid biosynthesis.	5.00/29.54	6.45/84.11	82	11
10	Hypothetical protein	OAL35951.1	*Fonsecaea nubica*	—	4.50/29.00	4.38/29.43	115	2
11	Isochorismatase hydrolase	KRH93850.1	*Verticillium dahlia*	Degrade Isochorismate, precursor of salicylic acid biosynthesis.	5.83/53.45	6.45/84.11	90	11
12	Hypothetical protein	XP_008716200.1	*Cyphellophora europaea*	—	7.25/36.00	5.13/344.20	84	15
13	Hypothetical protein	ORX65063.1	*Linderina pennispora*	—	6.15/45.40	10.18/393.28	100	25
14	Cys4P	AJR00164.1	*Saccharomyces cerevisiae*	Involved in Cystathionine biosynthesis.	8.13/36.15	6.25/56.08	82	15
15	Hypothetical protein	ODQ74899.1	*Lipomyces starkeyi*	—	8.25/54.50	8.20/136.32	81	13

**Figure 3:**
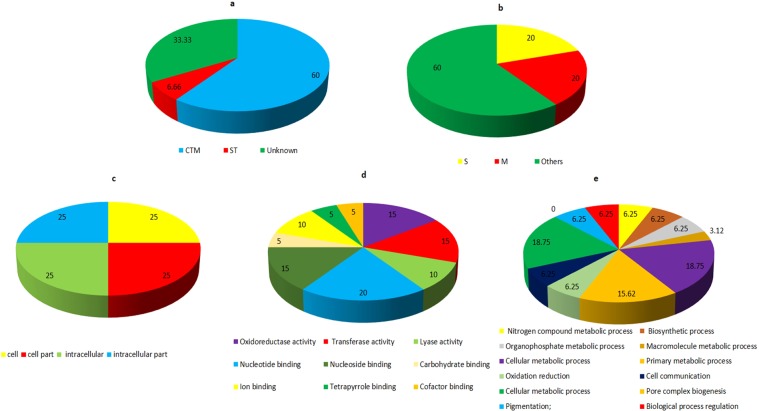
Functional annotation and subcellular localization of the proteins identified from TiK isolate mycelial proteome (**a**) Classification of the identified proteins through COG classification (**b**) Subcelluar localization of the identified proteins using TargetP; Gene Ontology based classification of the identified protein into distinct categories as cellular component (**c**), molecular function (**d**) and biological process (**e**).

The functional analysis of protein spots was carried out using Uniprot and InterproScan database. The identified proteins were classified into metabolic pathways through the COG classification. Carbohydrate transport and metabolism (CTM) related proteins comprises 60% of the total classified proteins. About 6.66% of the identified proteins belong to signal transduction (ST) classification and 33.33% of the protein were found to be hypothetical (Fig. 3a). The subcellular localization of the identified proteins were mainly categorized into other location (60%). While 20% proteins each were found to be component of the secretory pathway and mitochondria (Fig. 3b). On the basis of Gene ontology (GO), fifteen proteins varying in their expression in the presence of host factor, were classified into different categories such as molecular function, cellular component and biological process. Majority of the proteins in the molecular function category showed nucleotide binding ability (20%) followed by oxidoreductase (15%) and transferase (15%) activity (Fig. 3d). The GO analysis for biological process revealed that most of the proteins were involved in cellular metabolic process (18.75%) and primary metabolic process (15.62%) (Fig. 3e). 25% each of the cell, cell component, intracellular and intracellular component were found in cellular component (Fig. 3c).

#### Proteins in the suppression of plant defense response signalling may aid in overcoming host resistance

The defense responses of host plant against the fungal pathogens are tightly regulated through a complex network of signal transduction pathways^27^. Salicylic acid is an important factor responsible for the activation of plant defense responses against a broad range of pathogens. In plants, salicylic acid biosynthesis occurs via isochorismate and phenylpropanoid pathways. Interestingly, the protein identified in spot 3 (Fig. 2; Table 1) exhibited homology with salicylate hydroxlase, that belong to the oxidoreductases family and converts salicylic acid into catechol. The three spots, namely 7, 9 and 11 (Fig. 2; Table 1) were identified as isochorismatase hydrolase that catalyzes the conversion of isochorismate to 2,3-dihydroxybenzoate and pyruvate^28^. This inhibits the salicylic acid formation as isochorismate is a critical precursor for biosynthesis of salicylic acid in plants^29^.

#### Proteins in the carbohydrate metabolism and transport may be responsible for KB pathogenesis

Spot 1, found to be upregulated in the mycelial protein of TiK isolate grown in the presence of host factor, was identified as 1,4-benzoquinone reductase (QR) (Fig. 2; Table 1). This enzyme belongs to flavoprotein quinone reductases family that are involved in detoxifying intracellular quinones^30,31^. Identified proteins from spots 6 and 8 (Fig. 2; Table 1) showed homology with the enzyme involved in glyoxylate cycle, Isocitrate lyase (ICL) involved in cleavage of isocitrate into glyoxylate and succinate. The role of ICL in fungal pathogenesis has been reported in rice blast pathogen *Magnaporthe grisea*^32,33^ and anthracnose pathogen *Colletotrichum lagenarium*^34^.

#### Other identified proteins

Spot 5 (Fig. 2; Table 1) was identified as S-adenosylmethionine (SAM) synthetase which is a highly conserved protein and catalyze the SAM biosynthesis which is a major methyl group donor. Overexpression S-adenosylmethionine synthetase encoding gene, SasA in *Aspergillus nidulans* greatly affects the development, stress response and secondary metabolism^35^. In bacterium *Bacillus subtilis*, overproduction of SAM synthetase resulted in increased spontaneous sporulation^36^.

### Comparative secretome analysis of *T*. *indica* TiK isolate grown in the presence and absence of host extract

**Figure 4:**
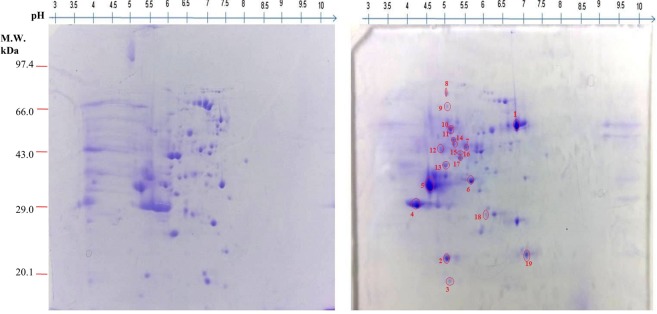
2-DE CBB stained gel of secretory proteins from *Tilletia indica* TiK isolate (**a**) cultured in the presence of host extract (**b**) cultured in the absence of host extract. Proteins were separated on 7 cm linear gradient IPG strips pH 3–10 and 12% polyacrylamide gel. Proteins spots exhibiting upregulation/differential regulation in TiK isolate (in the presence of host extract) are encircled red. Representative 2-DE gels are shown in Supplementary Figs S3 and S4.

**Figure 5:**
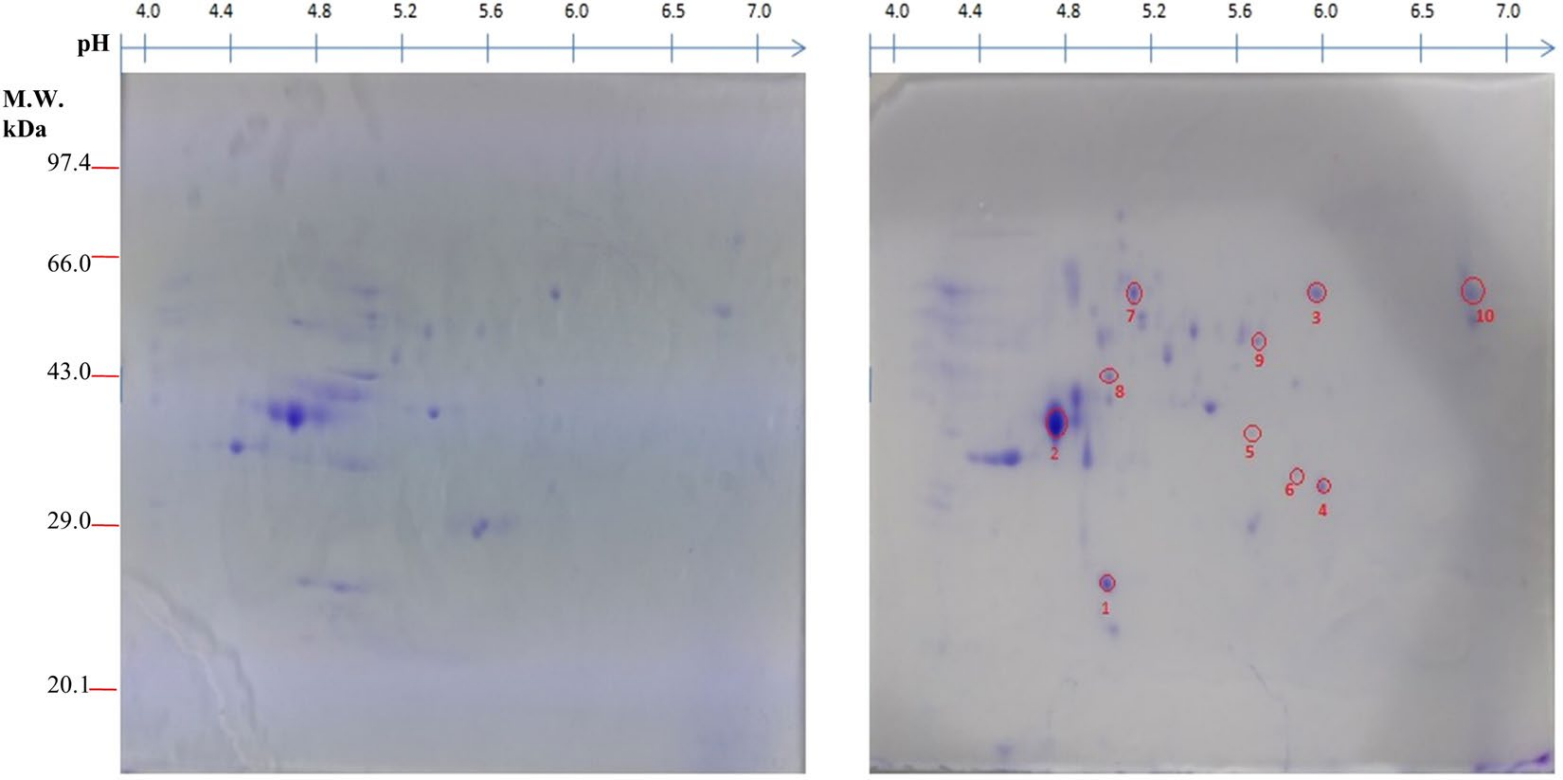
2-DE CBB stained gel of secretory proteins from *Tilletia indica* TiK isolate (**a**) cultured in the presence of host extract (**b**) cultured in the absence of host extract. Proteins were separated on 7 cm linear gradient IPG strips pH 4–7 and 12% polyacrylamide gel. Proteins spots exhibiting upregulation/differential regulation in TiK isolate (in the presence of host extract) are encircled red. Representative 2-DE gels are shown in Supplementary Figs S5 and S6.

The proteome maps (pH 3–10 and pH 4–7) of the secretome extracted from the TiK isolate grown in the presence and absence of host extract were obtained (Figs 4 and 5; Supplementary Figs S3–S6**)**. The comparative proteomic analysis of the secretome isolated from the TiK isolate cultured in the absence and presence of host extract revealed variations between the protein spots. The spots which were consistently found in three replicate gels were chosen further analysis by tandem mass spectrometry. Nineteen protein spots (at pH 3–10) showed 2.0 fold change were considered to be significant statistically (p < 0.05). Ten spots (at pH 4–7) that showed 1.5 fold change were found to be significant statistically (p < 0.05). These protein spots identified by MALDI-TOF/TOF.

#### MALDI-TOF/TOF analysis and database search

The identification of upregulated and differentially expressed proteins were carried out by MALDI-MS/MS-MASCOT. Ten and five spots from secretome map pH 3–10 and pH 4–7 were identified as proteins having well known functions. Identified proteins from secretome map pH 3–10 and pH 4–7 have the theoretical molecular mass between 15 to 441 kDa and 13 to 265 kDa, respectively. Identified spots are encircled and numbered on the gels (Figs 4 and 5). Putative identity of proteins, matched peptides number and the protein score are shown in Tables 2 and 3.

**Table 2:** Identified up-regulated and differentially regulated secretory proteins (pH 3–10) in highly aggressive *Tilletia indica* isolate TiK grown in the presence of host extract by MALDI-TOF/TOF.

Spot	Protein	Accession	Organism	Function	Exper. pI/Mr	Theo. pI/Mr	MASCOT Score	No. of Peptides
1	Glycosyl hydrolase 30	OAJ15739.1	*Tilletia walkeri*	Possess endo-β-1,4-xylanase activity.	6.85/64.43	8.38/51.67	264	11
2	Hypothetical protein	XP_007345262.1	*Auricularia subglabra*	—	5.14/24.00	5.16/44.05	71	12
3	Peroxiredoxin	ORY18094.1	*Neocallimastrix californiae*	Reduces H_2_O_2_ and protect pathogen against host generated reactive oxygen species.	5.25/19.85	7.60/21.12	89	13
4	Aspartyl protease	OAJ21989.1	*Tilletia walkeri*	Degradation of host cell wall proteins and antifungal proteins.	4.25/29.08	5.82/41.92	171	10
5	Related to stress response protein rds1p	OAJ21332.1	*Neurospora crassa*	—	4.62/36.00	4.93/31.31	117	5
6	Hypothetical protein	XP_002487729.1	*Talaromyces stipitatus*	—	5.24/36.17	8.99/77.28	84	15
7	Hypothetical protein	KJK82765.1	*Metarhizium anisopliae*	—	5.56/48.34	7.22/440.83	124	38
8	Hypothetical protein	OAJ20939.1	*Tilletia walkeri*	—	5.06/81.72	4.75/94.02	111	16
9	Hypothetical protein	KFY26206.1	*Pseudogymnoascus spp*.	—	5.12/75.64	5.25/30.93	121	24
10	Dual specificity phosphatase	OQD63390.1	*Penicillium polonicum*	Phosphatase activity	5.54/54.50	6.01/38.22	86	9
11	Dual specificity phosphatase	OQD63390.1	*Penicillium polonicum*	Phosphatase activity	5.54/54.48	6.01/38.22	86	9
12	Hypothetical protein	KIK53675.1	*Gymnopus luxurians*	—	5.00/44.56	6.02/104.535	136	24
13	Hypothetical protein	OBT79903.1	*Pseudogymnoascus spp*.	—	5.12/40.00	7.24/71.06	89	16
14	Serine/threonine protein kinase	ENH85273.1	*Colletotricum orbiculare*	Kinase activity	5.20/48.50	5.92/70.80	105	11
15	Uncharacterized protein	KLO88873.1	*Fusarium fujikuroi*	—	5.20/48.43	10.71/15.57	90	9
16	Fungal specific transcription factor	OAQ74759.1	*Purpureocillium lilacinum*	Transcription factor -	5.50/43.03	5.92/58.63	83	10
17	Fungal specific transcription factor	OAQ74759.1	*Purpureocillium lilacinum*	Transcription factor	5.50/42.78	5.92/58.63	83	10
18	Hypothetical protein	KHE85708.1	*Neurospora crassa*	—	6.20/28.60	6.30/22.71	95	15
19	Tagatose bisphosphate aldolase	KIR55487.1	*Cryptococcus gattii*	Convert Tagatose-1,6- bisphosphate to glycerone phosphate	7.10/2412	5.14/31.72	71	6

**Table 3:** Identified up-regulated and differentially regulated secretory proteins (pH 4–7) in highly aggressive *Tilletia indica* isolate TiK grown in the presence of host extract by MALDI-TOF/TOF.

Spot	Protein	Accession	Organism	Function	Exper. pI/Mr	Theo. pI/Mr	MASCOT Score	No. of Peptides
1	Galactose oxidase	OAJ22541.1	*Tilletia walkeri*	Involved in fungal mediated reactive oxygen species (H_2_O_2_) generation	5.00/25.24	5.31/43.67	86	9
2	Hypothetical protein	OAJ06104.1	*Tilletia indica*	—	4.75/40.32	7.75/18.72	94	3
3	Hypothetical protein	XP_018753474.1	*Fusarium verticillioides*	—	6.00/62.45	5.66/49.92	68	13
4	Pyruvate dehydrogenase	AG087536.1	*Blastobotrys aristata*	Aid the pathogen adhesion to host tissues	6.08/30.85	8.51/14.35	82	6
5	Glycosyl hydrolase 30	XP_003068038.1	*Coccidioides posadasii*	Possess endo-β-1,4-xylanase activity.	5.76/40.00	6.06/123.71	87	12
6	Hypothetical protein	XP_013275226.1	*Rhinocladiella mackenziei*	—	5.92/35.32	9.87/118..94	85	19
7	Hypothetical protein	OAJ04365.1	*Tilletia indica*	—	5.18/62.48	8.87/13.42	152	27
8	Adventurous gliding protein Z	KHX42225.1	*Colletotricum simmondsii*	—	5.05/43.06	4.79/94.99	104	56
9	Aspartyl peptidase	XP_013240365.1	*Tilletiaria anomala*	Degradation of host cell wall proteins and antifungal proteins.	5.82/54.5	5.82/41.92	120	18
10	Hypothetical protein	OAJ03869.1	*Tilletia indica*	—	6.38/6.45	9.41/40.88	173	3

**Figure 6:**
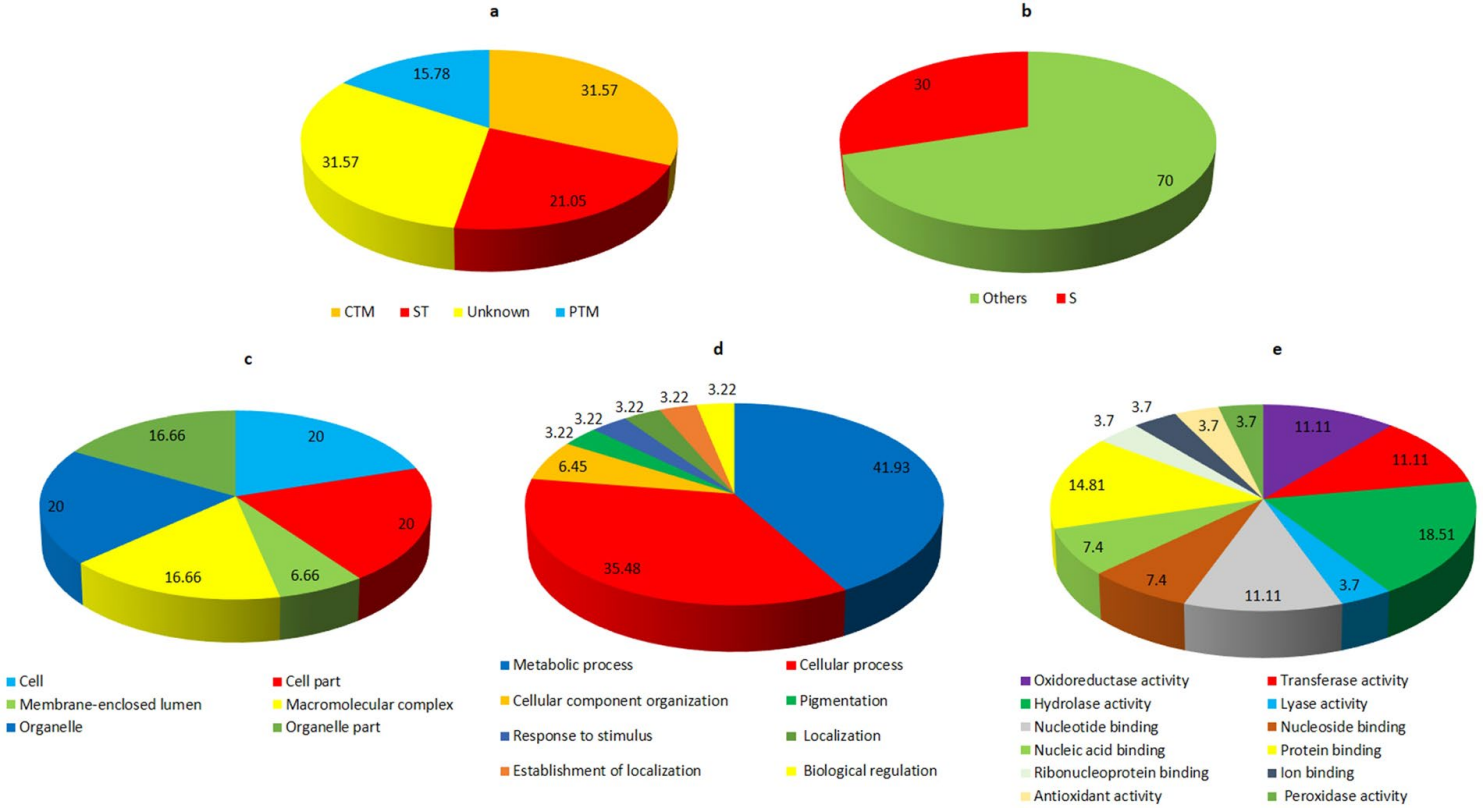
Functional annotation and subcellular localization of the proteins identified from TiK isolate secretome (**a**) Categorization of the proteins through COG classification (**b**) Subcelluar locations of the identified proteins using TargetP; Gene Ontology based classification of the identified proteins into distinct categories as cellular component (**c**), molecular function (**d**) and biological process (**e**).

Uniprot and InterproScan databases were used for the functional annotation of the identified protein spots. The identified proteins were categorized into various metabolic pathways using COG classification. About 31.57% of the identified proteins were found to be involved in Carbohydrate transport and metabolism (CTM). While the Signal transduction (ST) proteins comprises 21.05% of the total classified proteins followed by post translational modification (PTM) related proteins (15.78%) (Fig. 6a). The subcellular localization of the identified proteins showed that 30% of the proteins were localized in the secretory pathway while remaining were found to be present in any other location (Fig. 6b). According to GO classification, the identified proteins were assigned as different categories, namely molecular function, cellular component and biological process. Majority of identified proteins involved in the biological process exhibited hydrolase activity (18.51%) followed by protein binding ability (14.81%) (Fig. 6e). The GO classification for molecular function revealed that most of the proteins were involved in the metabolic process (41.93%) and cellular process (31.48%) (Fig. 6d). 16.66% each of the macromolecular complex and organelle part were dominant in the cellular component category (Fig. 6c).

#### Proteins in the carbohydrate metabolism and transport may be responsible for virulence of KB pathogen

The first enzyme of pyruvate dehydrogenase complex (PDC), pyruvate dehydrogenase is involved in converting pyruvate into acetyl CoA through the process known as pyruvate decarboxylation. Interestingly, the spot 4 (Fig. 5; Table 3) was identified as pyruvate dehydrogenase which is required for the generation of acetyl CoA that is utilized in the citric acid cycle. Pyruvate dehydrogenase is known to play both direct and indirect roles in fungal pathogenicity^37,38^.

#### Proteins responsible for fungal mediated ROS generation may contribute to *T*. *indica* virulence

The protein in spot 1 (Fig. 5; Table 3) corresponds to enzyme Galactose oxidase that belongs to oxidoreductase family. This free radical enzyme catalyzes the oxidation of set of primary alcohols, including galactose into aldehyde with simultaneous reduction of dioxygen to hydrogen peroxide (H_2_O_2_)^39^. In numerous phytopathogenic fungi, Galactose oxidase serve as potential pathogenicity factor by generating pathogen mediated ROS^39^.

#### Proteins with hydrolytic activities may aid in *T*. *indica* pathogenesis

Several plant pathogenic fungi secrete extracellular enzymes like proteases and cell wall degrading enzymes that serve as important virulence factors. Two spots, namely spot 5 (Fig. 4.46; Table 4.24) and spot 1 (Fig. 4; Table 2) were matched to Glycoside hydrolase (GH) protein 30 family that possess β-1, 4-endoxylanase activity. In the present study, aspartyl proteases has been identified in the spot 9 (Fig. 5; Table 3) and spot 4 (Fig. 4; Table 2) from the secretome of *T*. *indica* cultured in the presence of host extract, suggesting the probable role of aspartyl proteases as an important pathogenicity factor in *T*. *indica*.

**Table 4:** Validation of predicted three dimensional protein models by RAMPAGE, ProQ, DALI and ProFunc server.

Sequence	Template	Ramachandran plot	ProQ server		DALI server	Root Mean Square Deviation with template (A°)
		Residues in favored and allowed regions (%)	LG score	MaxSub	Z score	
TiBR	4la4A	100	3.929	0.328	36.5	0.76
TiGO	5a10A	96.5	2.926	0.261	55.1	0.78
TiPDH	1ni4A	98.7	2.759	0.217	58.9	0.30
TiGH30	5ngkA	97.4	3.332	0.19	51.7	1.09
TiPR	2p5A	98.3	3.268	0.349	27.1	0.55
TiAPs	2psgA	97.5	2.991	0.218	52.5	1.06

#### Proteins with antioxidant activity may protect *T*. *indica* against host generated ROS

Plant produce various reactive oxygen species (ROS) that changes the redox status of the host plant cells, thus, prevent the invasion of fungal pathogens by creating hostile environment and activating of many plant defences such as triggering the signal molecules, PR-proteins, phytoalexins. Interestingly, spot 3 (Fig. 4; Table 2) matched to peroxiredoxins that provide protection against host- generated ROS.

#### Other identified proteins

Some identified proteins in the secretome of *T*. *indica*, including fungal specific transcription factor domain (spot 16 and 17) (Fig. 4; Table 2) and stress response protein rds1p (spot 5) (Fig. 4; Table 2) were less characterized proteins of fungal origin.

### Candidate pathogenicity/virulence factors identification from *T*. *indica* genome

The homologs of pathogenicity/virulence factors, identified by tandem mass spectrometry from the mycelial proteins and secretome of TiK isolate grown in the presence of host extract, were searched against *T*. *indica* genome. It resulted in identification of putative homologs of candidate pathogenicity/virulence factors, namely 1,4-Benzoquinine reductase, Galactose oxidase, Pyruvate dehydrogenase, Glycoside hydrolase (GH30), Peroxiredoxin and Aspartate proteases from the *T*. *indica* genome. These sequences were designated as TiBR, TiGO, TiPDH, TiGH30, TiPR and TiAPs, respectively.

### Functional annotation of putative pathogenicity/virulence factors

#### TiBR

**Figure 7:**
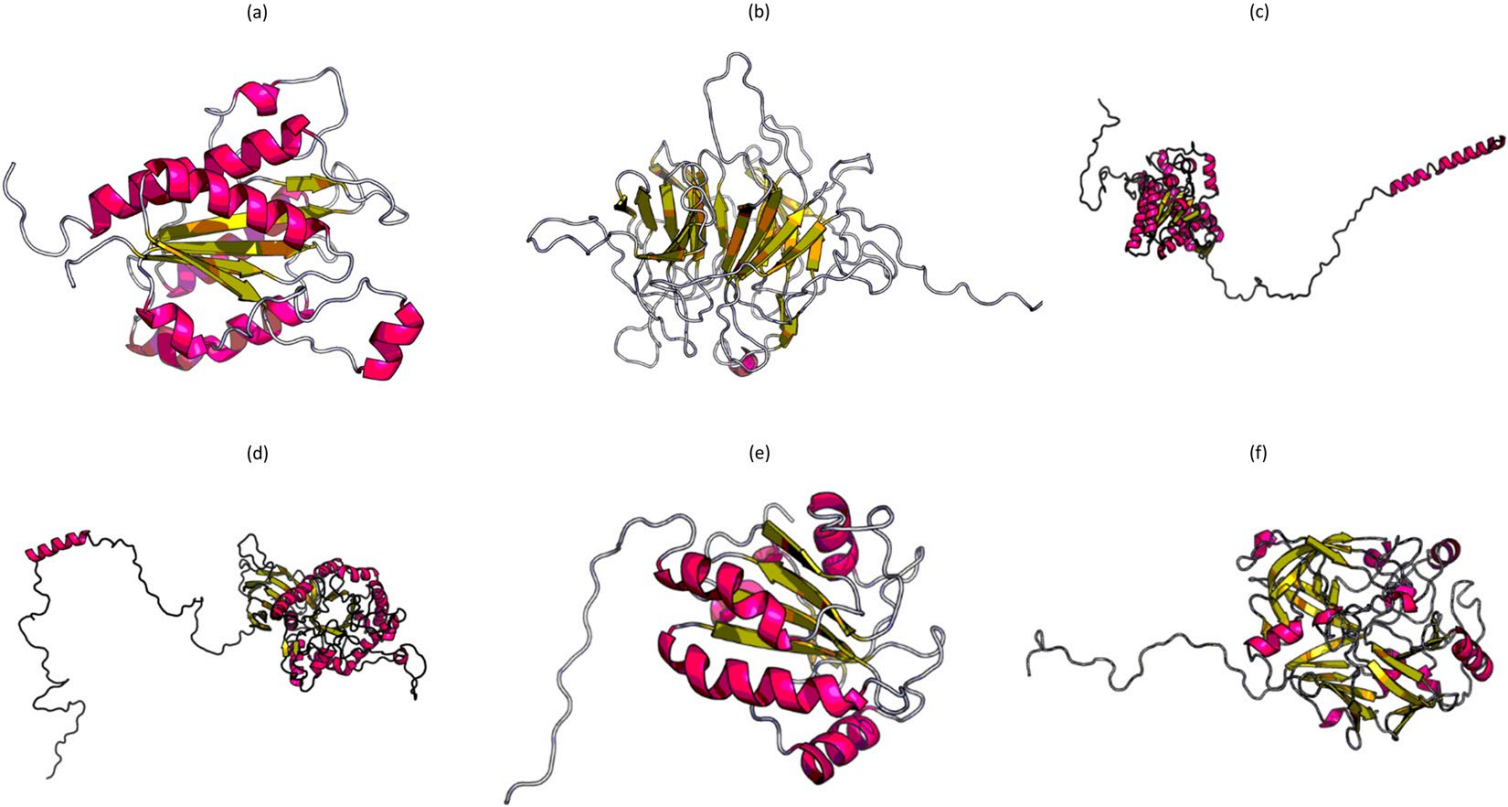
(**a**) Predicted three dimensional protein models for *Tilletia indica* candidate pathogenicity/virulence factors (**a**) TiBR (**b**) TiGO (**c**) TiPDH (**d**) TiGH30 (**e**) TiPR (**f**) TiAPs.

Domain analysis result suggested that this protein belongs to NADPH-dependent FMN reductase superfamily and has oxidoreductase activity (Table 5). This is consistent with the InterProScan result that also indicated the FMN-dependent NAD(P)H-quinone oxidoreductase activity of the protein (Table 5). PDB I.D. of the templates used for prediction of the 3-D structure of TiBR were 5mp4A, 2r96A and 4la4A. The p-value and score of the predicted three dimensional structure was 2.04e-06 and 164, respectively. The results suggested a very good quality protein model prediction (Fig. 7a).

**Table 5:** Sequence domains and structural motifs of the candidate pathogenicity/virulence factors identified in the mycelial and secretory proteins of *Tilletia indica*.

Protein Sequence	Domain	Structural motifs
TiBR	NADPH-dependent FMN reductase superfamily	Gly125-Glu127, Trp15-Val18; Asp175-Val178 and Gly205-Ala207
TiGO	Kelch-type beta propeller	Lys252-Ser254; Gly390-Asp392
TiPDH	TPP_enzymes superfamily, PDH_E1_alph_y motif	Gly205-Gly207; Gly225-Gln227; Asp424-Tyr426; Phe196- Arg198; Ser203-Gly205
TiGH30	Glycosyl hydrolase family 30 TIM-barrel domain	Gly418-Lys420
TiPR	thioredoxin_like superfamily with thioredoxin_like fold	Tyr87-Val89
TiAPs	Peptidase family A1	Ala228- Leu231

**Figure 8:**
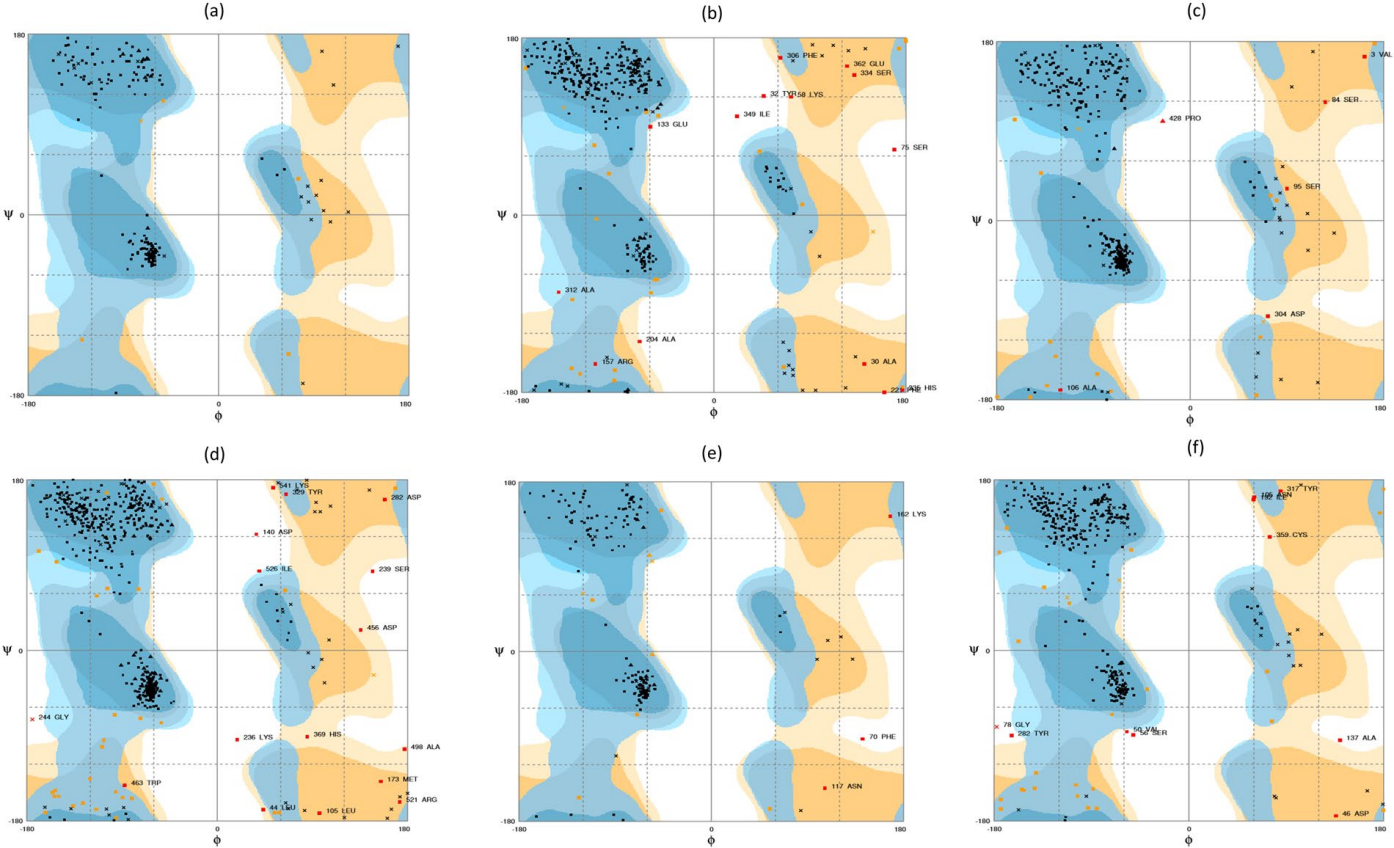
Ramachandran’s plot for three dimensional protein structure of candidate pathogenicity/virulence factors (**a**) TiBR (b) TiGO (**c**) TiPDH (**d)** TiGH30 (**e**) TiPR (**f**) TiAPs.

Ramachandran plot for the predicted TiBR protein structure showed that 97.6% and 2.4% residues were in the favoured as well as allowed region, respectively. Thus, altogether 100% of the residues fell in the favored and allowed regions. This suggested that the protein 3-D structure was of very high quality (Fig. 8a). The predicted protein structure has the RMSD of 0.76 A° wrt template 4la4A Table 4), which indicated same function. According to Server ProFunc prediction, NADPH-dependent FMN reductase with close resemblance to 1,4-Benzoquinone reductase. Structural motifs such as Gly125-Glu127, Trp15-Val18, Asp175-Val178 and Gly205-Ala207 were identified (Table 5). Similar results were obtained on DALI server (Z score = 36.5) that identified its homology with NAD(P)H oxidoreductase (quinone) (Table 4). The *in silico* analyses suggested that TiBR may probably be NAD(P)H-quinone oxidoreductase.

#### TiGO

The sequence-based analysis of putative TiGO suggested that it may be a putative galactose oxidase. Conserved domain analysis indicated that this protein belongs to Kelch_1 superfamily and has six copies of the kelch motif that forms a beta sheet, many such beta sheets associate to give rise to a beta propeller structure which is characteristic of enzyme galactose oxidase (Table 5). Same results were obtained by motif analysis using Interproscan and TiGO sequence showed to possess kelch motif and repeated sequence corresponding to 4-stranded antiparallel beta-sheet motif forms the repeat unit in a super-barrel structural fold (Table 5). This structure is characteristic of galactose oxidase enzyme. RaptorX software utilized the 3-D structures with PDB I.D. 5a10A, 5gq0A and 5gqtA as templates for TiGO protein model prediction. The p-value and score of the predicted three dimensional protein model was 1.55e-12 and 222, respectively (Fig. 7b).

Ramachandran plot for the predicted TiGO protein model showed that 90.8% and 5.7%, of the residues were in the favoured region and allowed region, respectively (Fig. 8b; Table 4). This indicated the prediction of a good quality protein model with 0.78 A° RMSD wrt the template 5a10A (Table 4). ProFunc analysis predicted the presence of kelch motif and structural motifs Lys252-Ser254 and Gly390-Asp392 (Table 5). TiGO showed a significant homology with protein galactose oxidase as predicted by DALI server (Z score = 55.1) (Table 4). *In silico* analyses strongly suggested that TiGO may acts as a galactose oxidase enzyme.

#### TiPDH

Putative TiPDH domain was conserved with TPP_enzymes superfamily that utilizes thiamine diphosphate (TPP) as a cofactor (Table 5). InterProScan results also indicated that TiPDH sequence belongs to Pyrv_DH_E1_asu_subgrp-y family, members of which are alpha subunit of the E1 component of pyruvate dehydrogenase (PDH) (Table 5). The E1 component of pyruvate dehydrogenase complex is involved in the conversion of pyruvate to acetyl-CoA. The sequence based analysis suggested that TiPDH possess pyruvate dehydrogenase activity. RaptorX used protein models with PDB I.D. 1ni4A, 4zmkA, 2ic9A and 3rguA as templates for the prediction of three dimensional protein structure. The predicted three dimensional protein model has a p-value and score of 4.56e-11 and 373, respectively, indicating a high quality protein model prediction (Fig. 7c).

Ramachandran plot suggested that 95.1% residues were in the favoured region. While 3.6% and 1.3% residues were in the allowed and outlier region (Fig. 8c; Table 4), indicating a good quality three dimensional protein structure prediction. RMSD of the predicted protein model wrt the template 1ni4A was 0.30 A° (Table 4). PDH_E1_alph_y motif was identified in the TiPDH sequence. Gly205-Gly207, Gly225-Gln227, Asp424-Tyr426, Phe196- Arg198 and Ser203-Gly205 were identified as structural motifs by Profunc server (Table 5). DALI indicated that predicted protein model has a functional similarity with α subunit of pyruvate dehydrogenase E1 component (Z score = 58.9) (Table 4). All the results suggested the probable pyruvate dehydrogenase activity of TiPDH.

#### TiGH30

Domain analysis suggested that TiGH30 belongs to Glyco_hydro_30 superfamily and has TIM-barrel domain (Table 4). InterProScan results also suggested its similarity with a member of Glycoside hydrolase family 30 (Table 4). RaptorX software utilized three dimensional protein structures with PDB I.D. 5ngkA, 2wnwA, 3k07A, 2gifA and 4k0eA as templates. The predicted three dimensional structure has the p-value of 7.71e-12 and score of 400 (Fig. 7d).

Ramachandran plot for the predicted three dimensional protein model showed that 92.4% and 2.6% residues were placed in the favoured and outlier region, respectively. While 5.0% residues were in the allowed region. About 97.4% of the residues were fell in the favored and the allowed categories as shown in Fig. 8d; Table 4. This indicated a high quality protein model prediction. TiGH30 structure exhibited resemblance with the template 5ngkA and 1.09 A° RMSD (Table 4). ProFunc server revealed that TiGH30 has Glycosyl hydrolase family 30 TIM-barrel signature with Gly418-Lys420 as structural motifs (Table 5). DALI search results indicated that predicted TiGH30 structure has functional similarity with the Glycoside hydrolase 30 (Z score = 51.7) (Table 4). These results suggested the possible β-1,4-xylanase activity of TiGH30.

#### TiPR

Conserved domain database showed that TiPR belong to thioredoxin_like superfamily having thioredoxin (TRX) - like fold (Table 5). The thioredoxin (TRX) - like fold in TiPR was also confirmed by InterProScan analysis, further suggesting that TiPR may be a thioredoxin protein (Table 5). 3-D protein model with PDB I.D. 2p5qA and 2rm5A, showing oxidoreductase activity, were utilized as templates for the prediction of three dimensional protein structure that has a p-value 2.75e-06 and score of 126 (Fig. 7e).

Ramachandran plot indicated that 93.3% and 1.7% of the residues were in the favoured and the outlier region while 5.1% residues were in the allowed region. Hence, 98.3% residues were in the favored and allowed regions as shown in Fig. 8e; Table 4). These results suggested the prediction of high quality three dimensional protein model that has an RMSD of 0.55 A° with respect to template 2p5qA (Table 4). Functional annotation showed that thioredoxin_like conserved motif was present in TiPR and Tyr87-Val89 as structural motif (Table 5). DALI server suggested its profound homology with thioredoxin (Z score = 27.1) (Table 4).

#### TiAPs

Domain analysis results indicated that TiAP belongs to Asp superfamily that comprises aspartyl proteases (Table 5). Interproscan analysis revealed that TiAPs belongs to Aspartic peptidase A1 family which has peptidases with bilobed structures with aspartic acid residue from each domain forming an active site (Table 5). 3-D models having PDB I.D. 2psgA, 1cmsA, 1uh7A, 3pepA and 1aptE were used as templates for three dimensional protein structure prediction with p-value 1.07e-16 and score 304, respectively (Fig. 7f).

According to Ramachandran plot, about 91.0% and and 6.5% residues fell in the favoured and allowed region whereas 2.5% of the residues were present in the outlier region (Fig. 8f; Table 4). Predicted three dimensional structure has an RMSD of 1.06 A° wrt template with PDB I.D. 2psgA (Table 4). Ala228- Leu231 was identified as structural motif by ProFunc server (Table 5). Similar results were obtained using DALI server that suggested a significant similarity of TiAP with aspartyl protease pepsin (Z score = 52.5) (Table 4). The bioinformatics analysis suggested that TiAPs may probably be aspartate protease.

## Discussion

Comparative analysis of mycelial proteome from TiK isolate grown in the absence and presence of host factor showed that the proteins involved in carbohydrate metabolism constitute the highest percentage (60%) in the proteins isolated mycelium grown the presence of host factor. These results strongly support the higher growth rate of TiK isolate when grown in the presence of host factor.

1,4-benzoquinone reductase was upregulated when the mycelium was cultured in the presence of host factor. The proteomic analysis of Douglas-fir response to laminated root rot pathogen *Phellinus sulphurascens* showed that 1,4-benzoquinone reductase of fungal origin was up-regulated during infection^40^. Another study demonstrated the importance of QR enzyme in the vanillin metabolism, which is one of the intermediate steps in lignin biodegradation^41^. This finding also suggests that lignin degradation may be an essential part of the infection strategy of KB pathogen. In the present study, spot 3 was identified as salicylate hydroxlase. The role of salicylate hydroxlase in pathogenicity has been reported in various fungi, including *U*. *maydis*, *Fusarium* spp., *Aspergillus nidulans*, *Trichosporon moniliiforme*, and *T*. *cutaneum*^42–46^. Three candidate salicylate hydroxylase genes have been identified in *U*. *maydis* that showed homology to *Pseudomonas* spp. *nahG*^42^. The high-throughput SOLiD-SAGE based comparative transcriptome study revealed that fungal transcripts corresponding to salicylate hydroxylase *nahG* gene was more abundant in *Epichloe festucae* - infected plant *Festuca rubra* than non-infected plant^47^. This gene from the bacterium *Pseudomonas putida* is responsible for conversion of naphthalene to pyruvate and acetaldehyde^48^. Salicylic acid, formed as an intermediate during naphthalene degradation is converted by NahG into catechol. The role of salicylate hydroxylase in degradation of plant-produced salicylic acid may be a possible mechanism by which *T*. *indica* evade the plant defense responses.

In phytopathogenic soilborne pathogen *V*. *dahliae*, isochorismatase hydrolase was found to be differentially expressed in the proteome of highly aggressive isolate^49^. Soanes *et al*.^28^ reported that the presence of isochorismatase motif containing proteins in the secretome of five types of plant pathogens, while protein with such motif were absent in the secretome of non-pathogenic ascomycete fungi. In *T*. *indica*, isochorismatase hydrolase (spot 7, 9 and 11) may act as suppressor of host plant defences and aid in overcoming the host resistance.

During infection of rice blast fungus *Magnaporthe grisea*, the expression of ICL gene was upregulated in mycelia, appressoria and hyphae. ICL gene deletion mutant showed significant reduction in cuticle penetration and appressorium formation, resulting in overall decreased fungal virulence^32,33^. icl1 gene expression was also observed during infection of *Leptosphaeria maculans* on *Brassica napus*. ICL deletion mutant exhibited the reduced pathogenicity of pathogen on cotyledons, lower germination rate of pycnidiospores and limited hyphal growth. The reduced fungal pathogenicity was due to inability to use the carbon sources of the host plant^50^. Studies by Asakura *et al*.^34^ demonstrated that ICL gene is required during the early stage anthracnose pathogen *Colletotrichum lagenarium* infection as icl1-deficient mutant were not able to form the penetrating hyphae on cotyledons. Similarly, the identified ICL protein (spot 6 and 8) may be also have a role in KB pathogenensis.

Comparative analysis of secretome from TiK grown in the absence and presence of host factor resulted in identification of various pathogenicity factors such as pyruvate dehydrogenase, galactose oxidase, GH 30, aspartyl proteases, peroxiredoxins. Spot 4 identified as pyruvate dehydrogenase has a direct function in pathogen adhesion to the host cells. For instance, in bacterium *Lactobacillus plantarum*, pyruvate dehydrogenase is proposed to aid the adhesion of pathogen to the host tissues^37^. Indirectly, it provides acetyl CoA for citric acid or TCA cycle by catalyzing the irreversible conversion of pyruvate into acetyl CoA. The TCA cycle results in formation of malate which then converted by malate dehydrogenase into oxaloacetate which is a well characterized pathogenicity factor in *B*. *cinerea* and *S*. *sclerotiorum*^38,51–54^. Galactose oxidase (spot 1) secreted by many fungal pathogens such as *Fusarium graminearum*, *U*. *maydis* and *Aspergillus* spp. function as a free radical enzyme in extracellular space and protect the pathogens from host plant defences by generation of reactive oxygen species such as H_2_O_2_. Moreover, studies suggested the involvement of Galactose oxidases in disrupting plant cell wall prior to fungal invasion^39^.

Plant pathogenic fungi produce a wide array of hydrolytic enzymes which are required for fungal pathogenesis. Among them, proteases and hydrolases are essential for the growth and survival of the pathogens. Upon encounter with specific host plant, phytopathogenic fungi produce plethora of cell-wall-degrading and proteases to break down the plant cell wall polymers, thereby facilitate the fungal penetration into the host tissues^55^. Phytopathogenic fungi such as *Magnaporthe oryzae*, *Cochliobolus sativus*, *Fusarium oxysporum*, *S*. *sclerotiorum*, *Verticillium dahliae*, secrete β-1,4-endoxylanases that are capable of cleaving the xylan (an important hemicellulose polymer present in plant cell walls) into short oligomers^55^.

Fungal proteases may target the proteins in the plant plasma membrane, increasing its permeability and thus play significant role in pathogenesis^56^. Moreover, the products (polypeptides) released by the proteolytic activity of extracellular proteases of plant pathogenic fungi may act as elicitors or damage-associated molecular pattern), that are eventually recognized by specific host cell receptors and triggers a series of signaling events, including activation of MAPK signalling pathway, production of protease inhibitors, pathogenesis related proteins and antimicrobial peptides against the proteases^57^. Many protease-deficient fungal pathogens were found to be non-pathogenic. These results suggested the role of proteases in different stages of fungal pathogenesis, including adhesion^58–61^. Among the proteases, the role of aspartyl proteases has been well characterized in fungal pathogenicity. In *B*. *cinerea*, causal agent of gray mold disease, aspartic protease serve as a key pathogenicity factor required during initial stage of infection^61^. Recent studies by ten Have *et al*.^61^ suggested the importance of aspartyl proteases as key pathogenicity factors in fungus *B*. *cinerea*. Li *et al*.^62^ carried out the proteomic analysis of *B*. *cinerea* secretome under different pH conditions. Thirteen spots were identified as aspartate protease, of which eleven were differentially expressed at pH 4.

Phytopathogenic fungi produce many reactive oxygen species (ROS) scavenging enzymes for detoxifying host generated ROS. Among these enzymes, ubiquitous family of peroxidases, Peroxiredoxins catalyzes the reduction of H_2_O_2,_ various hydroperoxides (ROOH) into the alcohols^63^. Peroxiredoxins are essential for virulence of some fungal pathogens such as *C*. *neoformans*^64^. Similarly, peroxiredoxins may act as potential virulence factor in *T*. *indica*.

## Conclusion

Despite being economically significant fungal pathogen, the knowledge regarding the molecular mechanisms of KB pathogenesis is at infancy. To date, information on the pathogenicity/virulence factors induced in pathogen *T*. *indica* by its host wheat is unavailable. In the present study, the modulation in the protein profile of mycelia proteins and secretome from TiK isolate cultured in the absence and presence of host factor was analyzed using mass spectrometry based proteomics approach. The identified proteins are involved in suppression of host defense responses (salicylate hydroxylate, isochorismatase hydrolase), lignin degradation of plant cell wall (1, 4- benzoquinone reductase), penetration (isocitrate lyase), adhesion of pathogen to host tissues (pyruvate dehydrogenase), pathogen mediated reactive oxygen species generation (Galactose oxidases), hydrolytic enzymes (Glycoside hydrolase family 30, Aspartate proteases), detoxification of host generated reactive oxygen species (peroxiredoxins). Complementation of proteomic and genomic analysis resulted in identification of several candidate pathogenicity/virulence factors in *T*. *indica* induced in the presence of host wheat. The identified pathogenicity/virulence factors may be utilized as biomarkers for KB resistant wheat variety screening, development of fungicides and sensitive diagnostics for rapid on- site KB detection.

## Supplementary information

Complementary Proteomics, Genomics approaches identifies potential pathogenicity/virulence factors in Tilletia indica induced under the influence of host factor
