# Supplementary material for: Complementary Proteomics, Genomics approaches identifies potential pathogenicity/virulence factors in Tilletia indica induced under the influence of host factor
Source: Sci Rep. 2019 Jan 24;9:553. doi: 10.1038/s41598-018-37810-1 (PMC6346058; doi:10.1038/s41598-018-37810-1)
Supplement: Supplementary file 1 — Complementary Proteomics, Genomics approaches identifies potential pathogenicity/virulence factors in Tilletia indica induced under the influence of host factor [file 41598_2018_37810_MOESM1_ESM.pdf]

# Complementary Proteomics, Genomics approaches identifies potential pathogenicity/virulence factors in *Tilletia indica* induced under the influence of host factor

**Vishakha Pandey[1], Atul Kumar Gupta[1], Manoj Singh[1], Dinesh Pandey[1] and Anil Kumar*[1]**

Author Affiliations

1 Department of Molecular biology and Genetic Engineering, G.B. Pant University of Agriculture and Technology , Pantnagar, Uttarakhand India.

*Corresponding author:

E-mail: anilkumar.mbge@gmail.com, ak_gupta2k@rediffmail.com.

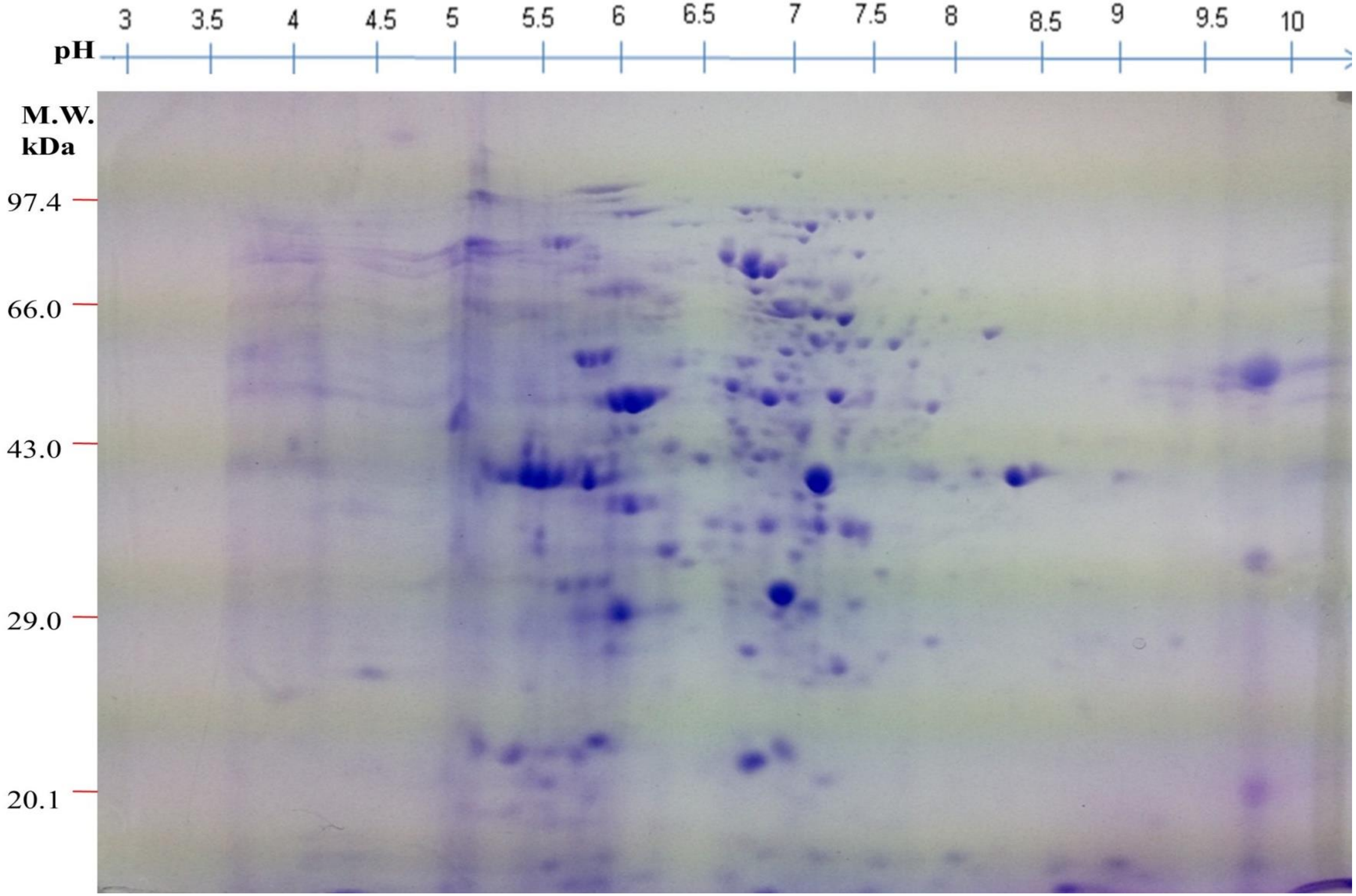


**Figure S1: Representative Coomassie Brilliant Blue (CBB)-stained two-dimensional electrophoresis (2-DE) gel image (pH 3-10) of proteins extracted from mycelia of *T. indica* TiK isolate (at 21 days of growth) cultured in the absence of host extract The MW (kDa) and pI scales are indicated.**

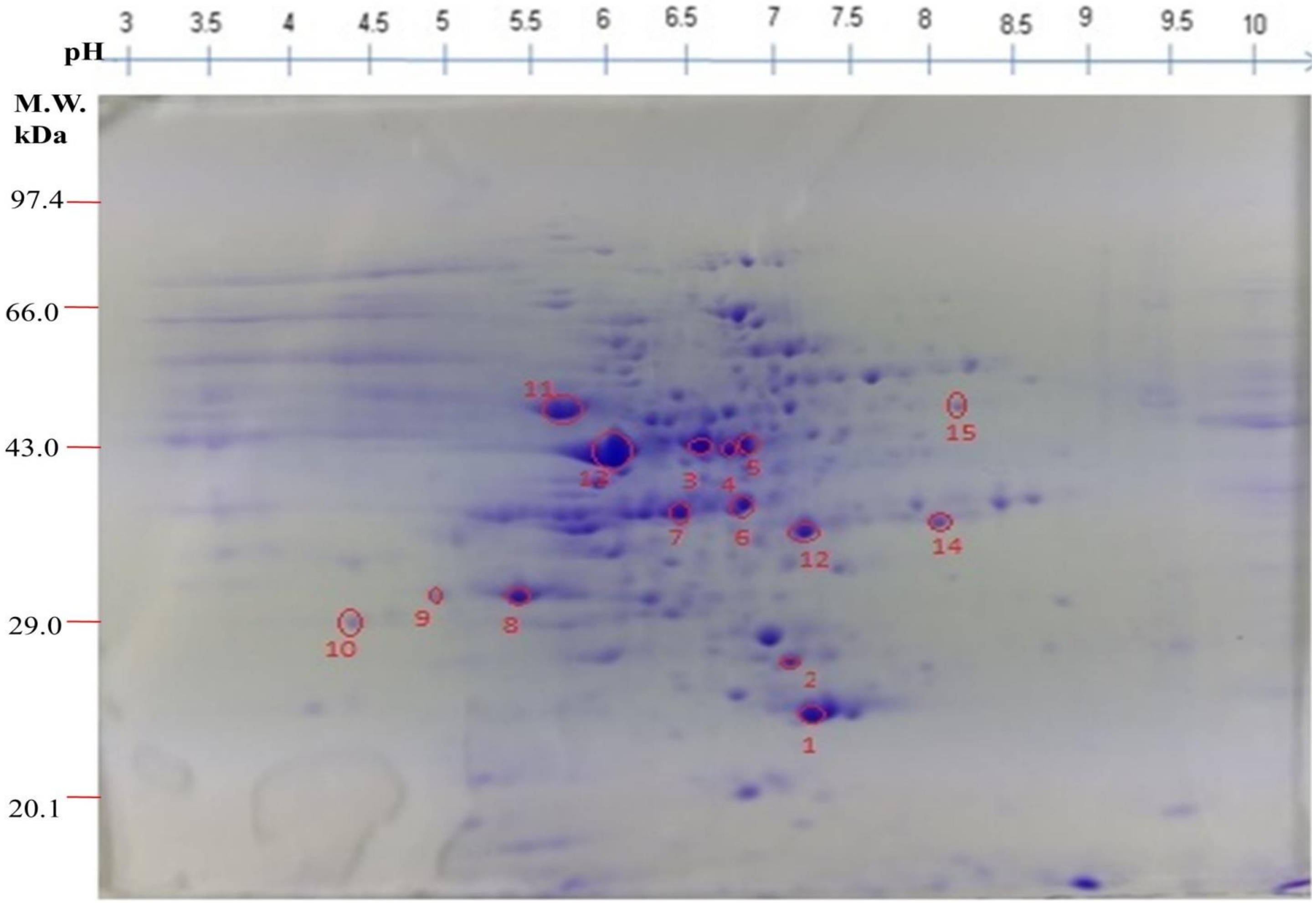


**Figure S2: Representative Coomassie Brilliant Blue (CBB)-stained two-dimensional electrophoresis (2-DE) gel image (pH 3-10) of proteins extracted from mycelia of *T. indica* TiK isolate (at 21 days of growth) cultured in the presence of host extract The MW (kDa) and pI scales are indicated.**

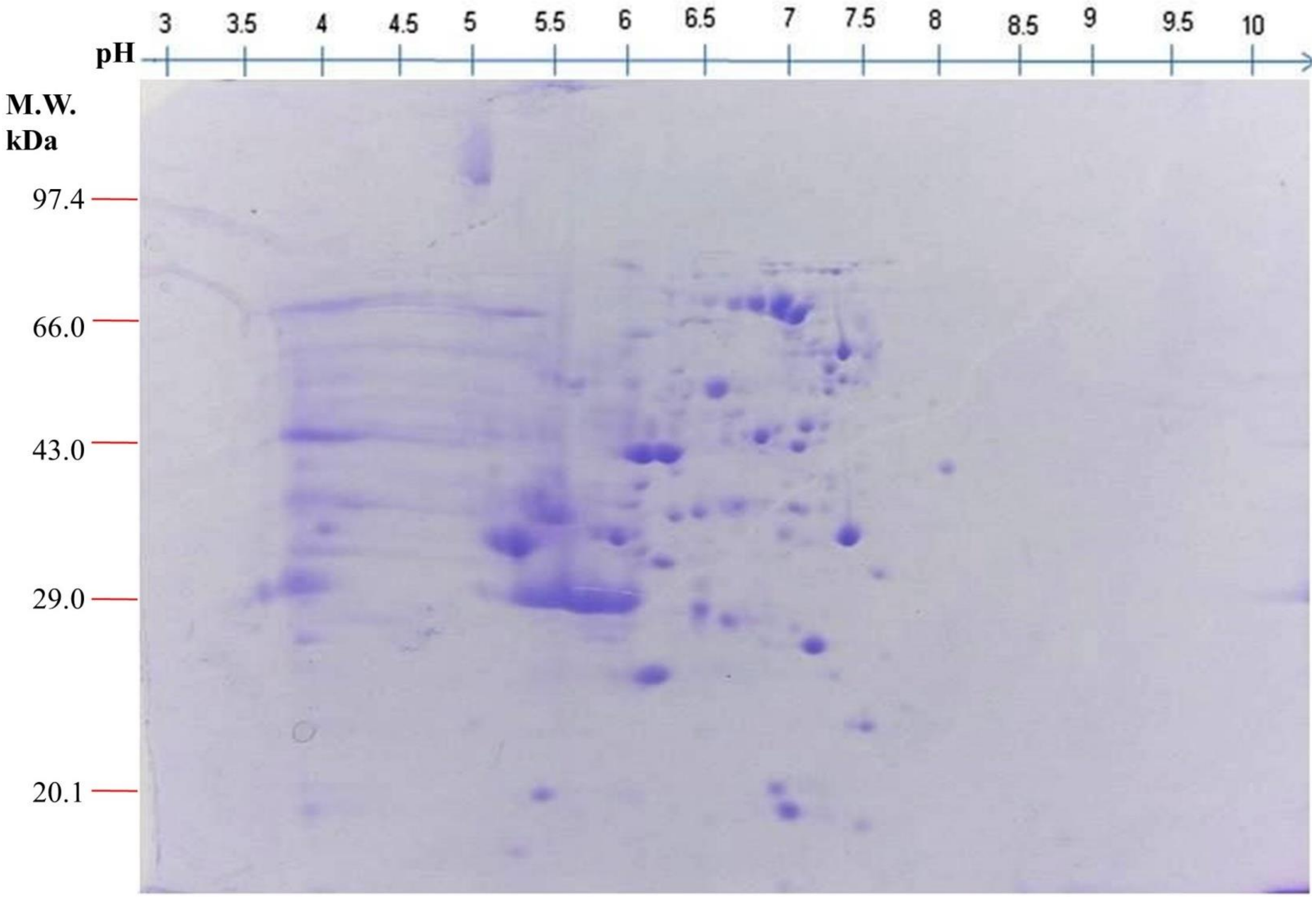


**Figure S3: Representative Coomassie Brilliant Blue (CBB)-stained two-dimensional electrophoresis (2-DE) gel image (pH 3-10) of proteins extracted from secretome of TiK isolate cultured in the absence of host extract . The MW (kDa) and pI scales are indicated.**

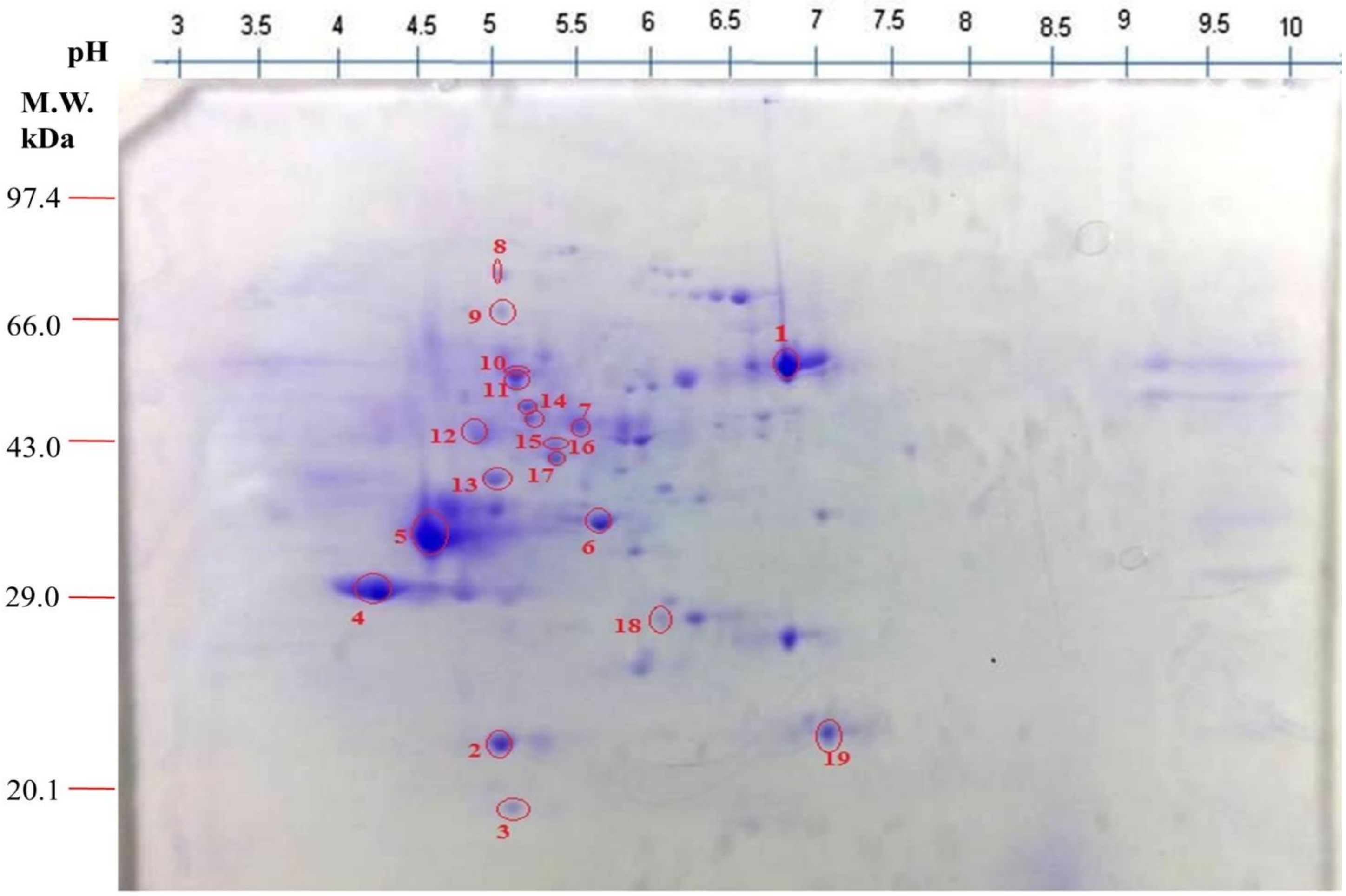


**Figure S4: Representative Coomassie Brilliant Blue (CBB)-stained two-dimensional electrophoresis (2-DE) gel image (pH 3-10) of proteins extracted from secretome of TiK isolate cultured in the presence of host extract . The MW (kDa) and pI scales are indicated.**

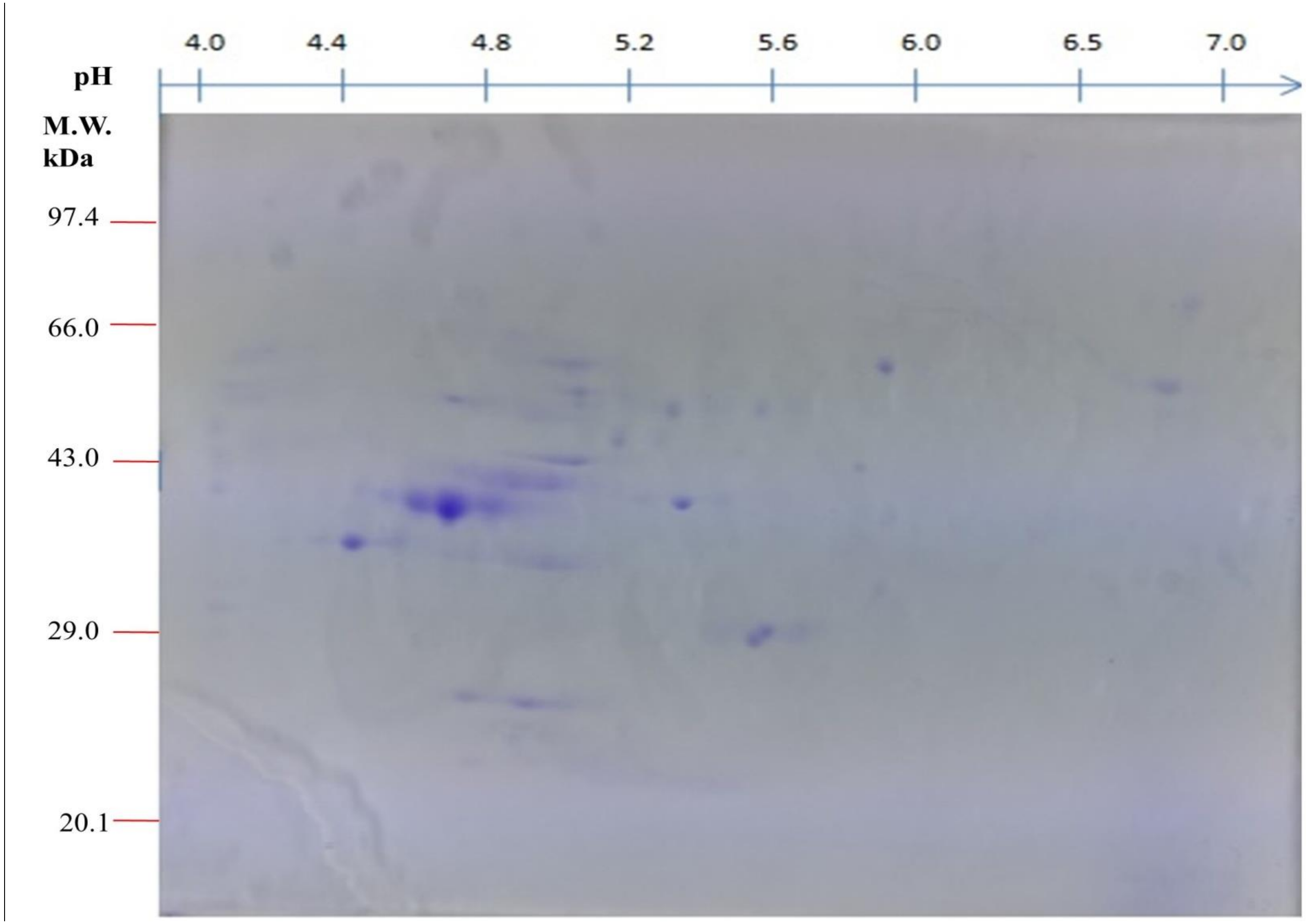


**Figure S5: Representative Coomassie Brilliant Blue (CBB)-stained two-dimensional electrophoresis (2-DE) gel image (pH 4-7) of proteins extracted from secretome of *T. indica* TiK isolate cultured in the absence of host extract. The MW (kDa) and pI scales are indicated.**

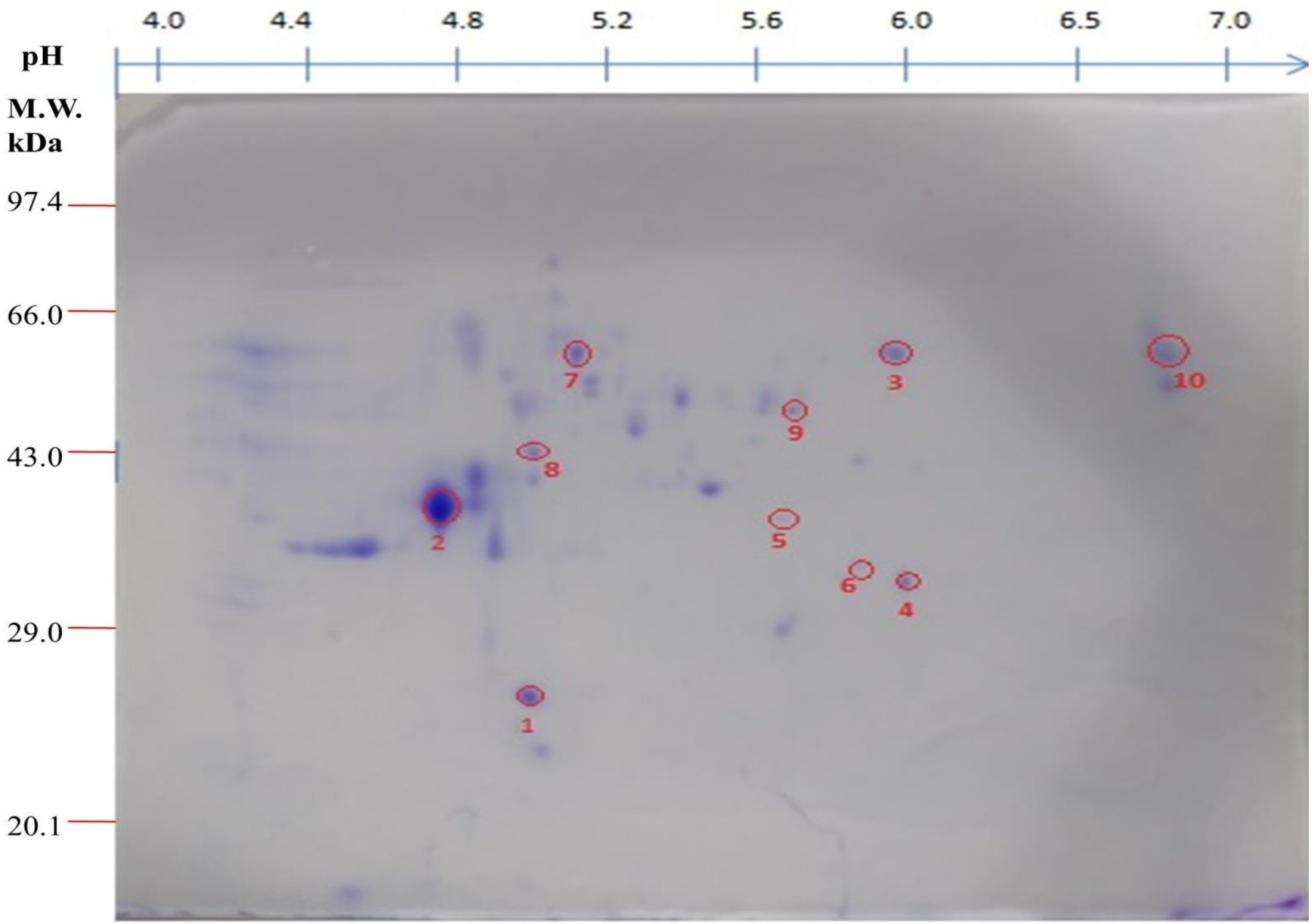


**Figure S6: Representative Coomassie Brilliant Blue (CBB)-stained two-dimensional electrophoresis (2-DE) gel image (pH 4-7) of proteins extracted from secretome of *T. indica* TiK isolate cultured in the presence of host extract. The MW (kDa) and pI scales are indicated.**
